# Understanding of Immune Escape Mechanisms and Advances in Cancer Immunotherapy

**DOI:** 10.1155/2022/8901326

**Published:** 2022-03-31

**Authors:** Nasrin Aktar, Chen Yueting, Muhammad Abbas, Hajra Zafar, Ana Cláudia Paiva-Santos, Qin Zhang, Tingting Chen, Moudud Ahmed, Faisal Raza, Xiaohui Zhou

**Affiliations:** ^1^Department of Clinical Pharmacy, School of Basic Medicine and Clinical Pharmacy, China Pharmaceutical University, Nanjing, Jiangsu 211198, China; ^2^Riphah Institute of Pharmaceutical Sciences, Riphah International University, Islamabad, Pakistan; ^3^School of Pharmacy, Shanghai Jiao Tong University, Shanghai, 200240, China; ^4^Department of Pharmaceutical Technology, Faculty of Pharmacy, University of Coimbra, 3000-548 Coimbra, Portugal; ^5^REQUIMTE/LAQV, Group of Pharmaceutical Technology, Faculty of Pharmacy, University of Coimbra, 3000-548 Coimbra, Portugal

## Abstract

Tumor immune escape has emerged as the most significant barrier to cancer therapy. A thorough understanding of tumor immune escape therapy mechanisms is critical for further improving clinical treatment strategies. Currently, research indicates that combining several immunotherapies can boost antitumor efficacy and encourage T cells to play a more active part in the immune assault. To generate a more substantial therapeutic impact, it can establish an ideal tumor microenvironment (TME), encourage T cells to play a role, prevent T cell immune function reversal, and minimize tumor immune tolerance. In this review, we will examine the mechanisms of tumor immune escape and the limits of tumor immune escape therapy, focusing on the current development of immunotherapy based on tumor immune escape mechanisms. Individualized tumor treatment is becoming increasingly apparent as future treatment strategies. In addition, we forecast the future research direction of cancer and the clinical approach for cancer immunotherapy. It will serve as a better reference for researchers working in cancer therapy research.

## 1. Introduction

**Figure 1:**
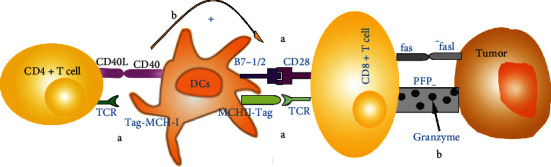
Mechanism of T cells in immunomodulation in cancer therapy. (a) T cells activate the first signal: CD4^+^ T cells bind to MHC-I signal carrying tumor antigen, and CD8^+^ T cells bind to MHC-I signal carrying tumor antigen; T cells activate the second signal: CD28 molecule binds to B7 on the surface of antigen-presenting cells. (b) CD4^+^ T cells, as helper T cells, enhance the antitumor effect mainly by stimulating the activation of CD8^+^ T cells; as cytotoxic cells, CD8^+^ T cells kill tumor cells mainly through Fas/FasL pathway and granule exocytosis pathway.

Cancer is one of the world's most lethal chronic diseases, and scientists have been eager to find a cure. Stutman established for the first time that immune system-mediated cytotoxicity is critical in preventing spontaneous tumor development. Researchers have discovered that effective tumor treatment modalities, such as radiation or chemotherapy, may be aided by the immune system [1]. Mature T cells in the thymus develop into CD4^+^/CD8^+^ single negative cells activated by costimulatory receptor and CD3^+^ T cell receptor (TCR) binary signals to increase antitumor activity. The cytotoxicity induced by CD8^+^ T lymphocytes can drive tumor cell death primarily through granule exocytosis and the Fas/FasL pathway [2, 3]. Wang et al. [4] recently demonstrated that CD8^+^ T lymphocytes stimulated by immunotherapy may raise ferritin specific lipid peroxidation (LPO) in tumor cells via IFN-, and that ferritin increase can contribute to immunotherapy's antitumor impact [5]. As a result, T cell-induced iron poisoning of tumor cells may be a possible method for tumor therapy. CD4^+^ T cells can improve dendritic cell (DC) ability to stimulate CD8^+^ T cell response via the CD40L/CD40 pathway [6] and encourage DCs to produce IL-2 to enhance CD8^+^ T cell antitumor immunological impact [7]. The mechanism diagram is shown in Figure 1. In the study of tumor immunotherapy, scientists discovered that it would employ a series of measures, including selective expression of tumor neoantigens (TNAs) and major histocompatibility complex (MHC) defects [8], to establish a local immunosuppressive microenvironment to induce T cells to express impotent, evade immune system monitoring, and promote tumorigenesis. It can also successfully resist chimeric antigen receptor (CAR) T cell treatment, tumor vaccines, and other immunotherapy methods [9, 10] that boost T cell immune response, evade immune system surveillance, and make immunotherapy difficult. The immunological microenvironment of most tumors has been shown to be strongly suppressed, which is the major barrier to inducing immune-mediated killing of cancer cells. Although the research of tumor pathogenesis is still in its early stages, tumor immune escape has shown to be a barrier to translating theoretical understanding of tumor pathogenesis into clinical therapy [11]. Researchers are working hard to find therapeutic techniques that would disrupt the tolerance to tumor antigens. Since the food and drug administration (FDA) authorized Keytruda to treat melanoma in 2014, there has been a boom in scientific study on tumor escape immunotherapy. Despite tumor immunotherapy has traditionally focused on reducing tumor immune escape, researchers have shown that even blocking tumor immune escape targets might contribute to tumor growth over time, resulting in therapeutic failure. This has compelled researchers to reexamine the “gap” between immunotherapy and the mechanism of tumor immune escape, as well as develop novel immunotherapy techniques to slow tumor progression.

## 2. Tumor Immunotherapy Dilemma

**Figure 2:**
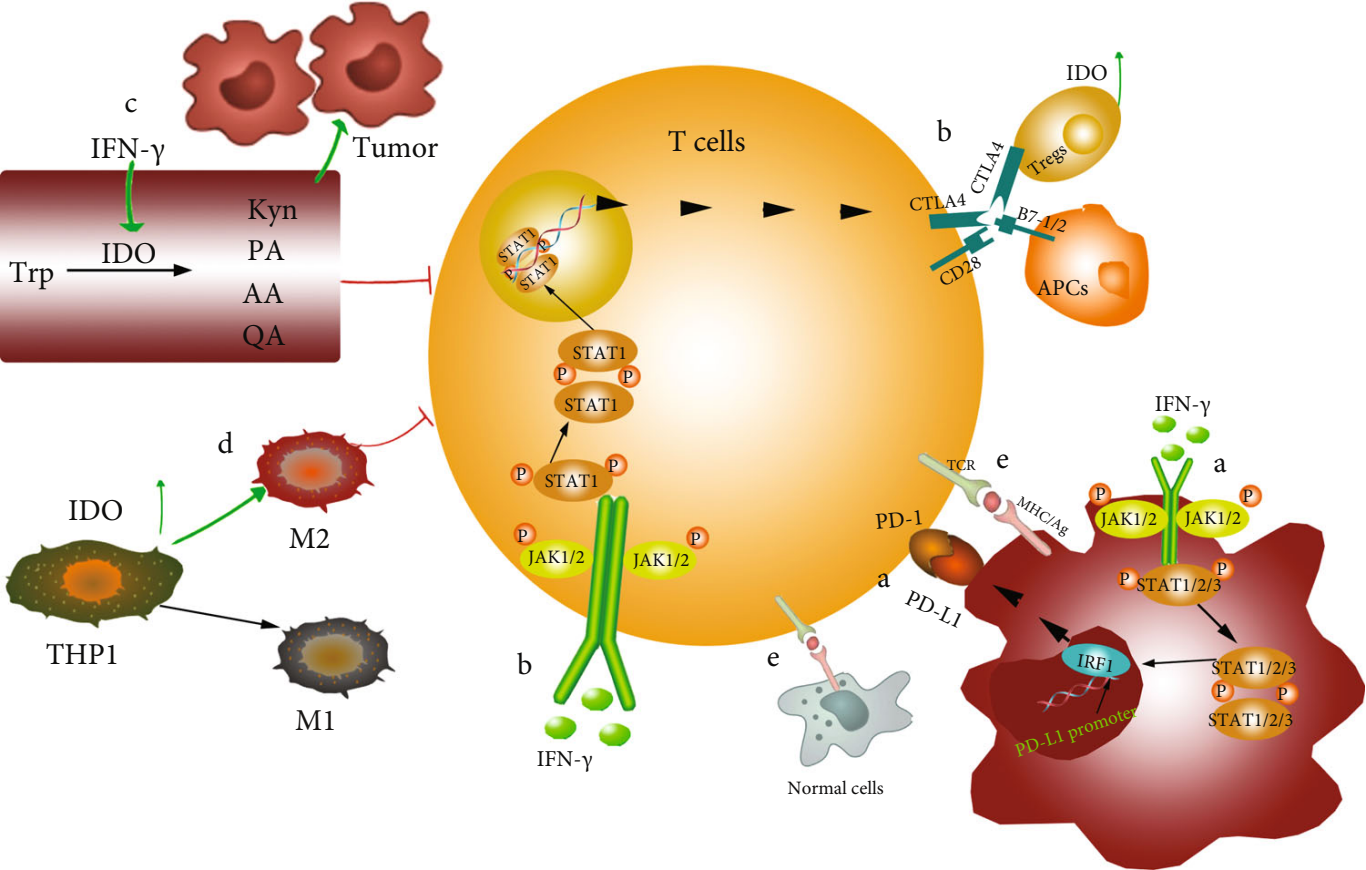
Multiple pathways of tumor immune escape mechanism. (a) IFN-*γ*--JAK1/JAK2-STAT1/2/3-IRF1 axis mainly regulates the expression of PD-L1 [13]. If the expression of IFN-*γ* increases, the expression of PD-L1 will increase. The PD-1/PD-L1 pathway was activated and induced T cell inactivation. (b) IFN-*γ* activates CTLA-4 expression through phosphorylation of Jak1/2-STAT1 and then competes with CD28 to bind to B7 [14] that inhibits T cell activation. (c) IDO suppresses the immune response by metabolizing tryptophan [15]. Its metabolites inhibit T cell activation and promote tumor proliferation. (d) IDO expression in THP-1 cells increased M2-type cell polarization [16]. M2-type macrophages inhibit the immune system and promote tumor proliferation and metastasis. (e) T cells combine with tumor-associated antigens expressed by tumor cells to exert an immune effect.

Numerous publications have already subjected the process of tumor immune escape. This review will concentrate on tumor immunotherapy's failure regarding tumor immune escape (Figure 2) with a brief discussion.

### 2.1. Selectively Express Immune Escape Targets

In general, an immunotherapy that boosts T cells' antitumor impact can destroy a large number of tumor cells in the early stages [9]. However, as demonstrated in Figures 2(a)–2(c), tumor cells can evade immune system detection via PD-1/PD-L1, CTLA4/B7, IDO, and other pathways, resulting in treatment failure. When a specific immunotherapy technique is utilized, the tumor detects an “autoimmune risk target” and reduces its expression [12]. Simultaneously, it will gradually express another “safe” target capable of evading immune system detection and completing tumor spread. For example, Majzner and MackKall [10] showed that long-term CAR-T cell therapy application may decrease target antigen expression, which has emerged as a key problem influencing the durability of CAR T cell treatment. At the moment, monotherapy dominates tumor immunotherapy, and the tumor is easily tolerable to this immunotherapy. As a result, one of the primary goals is to reduce tumor tolerance.

### 2.2. Low-Specific Immunotherapy

Cancer immunotherapy works by breaking immunological tolerance and stimulating T cells' immune response to tumor cells. T cell immunotherapy primarily targets tumor-associated antigens (TAAs), which can be found in both healthy tissues and tumor cells, as seen in Figure 2(e). As a result, whereas breaking T cell immune tolerance to TAAs is straightforward, it is equally easy to break immune tolerance to healthy tissues, resulting in immunological-related adverse responses [17, 18]. Research published in 2019 found that the combination of nivolumab and ipilimumab had therapeutic relevance in treating prostate cancer (PCa), although the operation was eventually discontinued owing to severe side effects (2019). As a result, minimizing immune-related adverse effects while maintaining antitumor immunological impact is a therapy option that researchers should investigate.

### 2.3. Individualized Limitations of Immunotherapy

At present, tumor immunotherapy mainly focuses on protocolized therapy, but different types of tumors and individuals and subtypes of the same tumor have other effects on the same immunotherapy [1]. This is mainly due to the unique TME and different escape targets of tumor expression. Therefore, expanding personalized therapy is incredibly important. First, new potential biomarkers need to be continuously explored to develop clinical applications and response rates. As shown in Figure 2(a), PD-1/PD-L1 binding induces T cell inactivation so that PD-L1 can be used as a biomarker. However, just a few biomarkers in clinical use require additional investigation. Second, TNAs produced by non-synonymous tumor mutations can be utilized as a novel target. T cells can not only attack tumor cells precisely, but they can also avoid harming healthy tissues. However, neoantigen-targeted immunotherapy research is still in its early stages. As a result, personalized immunotherapy remains restricted. Tumor immunotherapy has gradually become one of the main directions of tumor therapy. Researchers are investigating the current limitations of tumor immunotherapy: selectively express immune escape targets, low-specific immunotherapy, and individualized immunotherapy limitations; it is critical to gradually break through the limitations. Tumor immunotherapy in combination can diminish tumor tolerance and, to some extent, overcome immunotherapy failure caused by preferential expression of tumor escape targets. Tumor individualization and targeted treatment can boost antitumor immunity and minimize immune-related side effects. As a result, it can address the issue of immunotherapy's limited specificity. As a result, these two immunotherapy research approaches will be among the most important in the future, and they are the primary focus of this study.

## 3. New Directions of Tumor Immunotherapy

Antitumor immunotherapy is mainly based on the immune system, which T cells dominate. Beatty et al. [19] concluded that T cells activate and fully participate in the antitumor immunological response in three stages: (1) Inhibit the signal pathways in the tumor microenvironment (TME) that promote tumor growth and metastasis and provide a better immunological milieu for T cells to perform an immune role. (2) Induce T cell initiation and activation sufficiently to enable proper antigen presentation. (3) Immunological mechanisms that limit T cell functions are reversed, including immune checkpoint blockade in the maintaining T peripheral tolerance. Tumor immunogenicity and tumor microenvironmental immunophenotype may have an impact. The treatment method used in these three stages differs depending on the tumor and the individual. With the advancement of research into tumor immune escape and cancer features, immunotherapy has progressively reached a new stage of development. According to the above-mentioned summary of the present state of tumor immunotherapy, it is known that there is currently a need to reduce tumor tolerance by combining immunotherapy and specifically increasing patients' response rate to malignancies through tailored treatment. As a result, combination immunotherapy and customized tumor therapy may be the clinical tumor treatment approaches of the future.

### 3.1. Immune Checkpoint Blockade

Recent preclinical investigations on the impact of immune checkpoint inhibitors (ICIs) in several cancer cell lines [20] show that combination ICIs may outperform single antigens in terms of survival. Fan et al. [21] demonstrated that combining the CTLA-4 antibody with ICOS, an induced co-stimulator, may considerably increase the antitumor efficacy and immunosuppressive response in established mice melanoma and PCa models. As a result, combining various ICIs can improve patients' response rate and survival, which could be one of the novel immunotherapy strategies. Simultaneously, tumor patients' immunological distinctions should be considered so that tumor specificity plays an important role in immune checkpoint blocker therapy.

#### 3.1.1. Combined Application of PD-1/CTLA4 Antibody

Both PD-1/PD-L1 and CTLA-4 antibodies have shown substantial efficacy in treating malignancies on their own and have been authorized for marketing by the FDA. However, these antibodies are only effective in a subset of individuals, and the response rate is low (Rotte, Jin and Lemaire 2018). Larkin and colleagues' [22] clinical studies have shown that, as compared to single-drug therapy, patients in the nivolumab and ipilimumab combination treatment for melanoma had a 5-year overall survival rate of more than 50%. In comparison, individuals treated with nivolumab or ipilimumab had a 5-year overall survival rate of less than 50%. As a result, using the PD-1/CTLA-4 antibody in combination can increase patient response and survival. The FDA's approval of nivolumab-plus-ipilimumab for patients with metastatic melanoma, advanced renal cell carcinoma, and colorectal cancer who have MMR and MSI-H abnormalities underlines the therapeutic potential of combination treatment. The completed clinical trials and the outcome of immune checkpoint combination therapy are provided in (Table 1), and the ongoing clinical trials are mentioned in supplementary material (Table S1). Shi et al. [23] demonstrated that combining immune checkpoint blockade with adoptive T cell treatment (ACT) provided a more sustained antitumor response than monotherapy for melanoma. This work establishes an experimental foundation for potential immunotherapy treatments for cancers that include CTLA-4 and PD-1 dual inhibition. As a result, combination immunotherapy may become one of the most important study areas in the future.

Abbreviations Ipi: ipilimumab; Pac: paclitaxel; PFS: progression-free survival; Pla: placebo; ORR: objective response rate; Gem: gemcitabine; Cis: cisplatin; Pem: pemetrexed; Erl: Erlotinib^[1]^ participants have not been followed up long enough in the current database to estimate.

#### 3.1.2. Cancer Specificity Deals with PD-1/PD-L1

According to one research [24], only 10% to 30% of patients with indications are affected by anti-PD-1/PD-L1 antibodies, which is quite perplexing. [25] discovered that tumor cells produce PD-L1 exosomes, which dramatically suppress T cell activation. However, PD-L1 inhibitors in exosomes appeared to have a little inhibitory impact. The author examined the levels of PD-L1 mRNA and protein in PC3 (prostate cancer cell line) and SK-MEL-28 (melanoma cell line). The results indicated that PC3 secretes a high quantity of PD-L1 exosomes, which explains why PCa and melanoma respond differently to PD-1/PD-L1 antibody. Chen et al. [26] discovered that measuring the PD-L1 content of exosomes in patients with melanoma treated with pembrolizumab can reveal distinct antitumor immune states. To some extent, data suggests that exosome PD-L1 is a significant role in treatment failure. Poggio et al. [25] discovered that blocking exosome PD-L1 secretion may greatly improve the effect of PCa on the PD-1 antibody, thus it is possible that interfering with exosome PD-L1 secretion could improve the antitumor immune response. NSMASE2 (SMPD3) and RAB27A are important exosomal biogenesis enzymes. SMPD3 increases vesicle endosome germination, while RAB27A aids in the fusing of MVB and plasma membrane. As a result, by controlling these enzymes, PD-L1 exosomes can be intervened in vivo [25].

There are several predictors that may be used to assess whether a patient has reacted to the anti-PD-1/PD-L1 antibody, and different types of cancers respond differently to the predictors. The biomarker PD-L1 has been utilized to predict the good response rate of the PD-1 antibody in the treatment of malignancies. Melanoma cells produce PD-L1 exosomes, according to recent research. In melanoma patients, the levels of PD-L1 in exosomes before and after treatment with pembrolizumab may represent distinct antitumor immunity [26]. As a result, PD-L1 is appealing as a blood biomarker. However, PD-L1 is not a strong predictor of clinical response in lung squamous cell carcinoma [27]. Perhaps this phenomenon may be related to immunological variations in the TME and distinct tumor cell properties. However, these differences can be exploited to tailor specialized immunotherapy to individual tumor patients better. So far, the PD-1 antibody has not demonstrated a substantial benefit in PCa patients. Simultaneously, the expression of PD-1/PD-L1 in PCa tissues is debatable, and it may be connected to tumor features. Kwek and colleagues [28] showed that the PD-1 antibody has a longer survival time than high PD-1 expression in PCa.

Nevertheless, Abida et al. [29] reported that the use of the PD-1 antibody had more clinical relevance in PCa patients with microsatellite instability. Therefore Understanding the specificity of tumor biology might thus give a biomarker to broaden clinical applicability. The usage of matching antibodies to the patient response may be considerably increased based on the tumor-specific biomarkers discovered.

#### 3.1.3. Therapeutic Specificity

Nivolumab, in combination with ipilimumab, has been FDA authorized for the treatment of melanoma. However, only 25% of PCa patients respond to it, and it is still not utilized for the treatment of PCa owing to a significant number of adverse effects (2019). MDSCs have been linked to PCa immune escape, according to Lu and colleagues [30]. The immune checkpoint blocker coupled with MDSCs targeted therapy demonstrated significant antitumor response in the mouse tumor model, showing that tumor specificity is critical to the treatment approach and efficacy. There are now more than a handful of FDA-approved indications for PD-1/PD-L1 inhibitors for tumor therapy, although not all patients with tumor indications react to the treatment of inhibiting immunosuppressive molecules [31]. This is because cancer is unique to each individual. A potential research area might be how to cope with T cell depletion produced by a non-PD-1-dependent mechanism to allow the tumor to evade immune monitoring. Antigen-activated T cells require enhanced mitochondrial activity [32]. Kumar and colleagues [33] revealed that TME inadequate to supply the required energy for T cell activation was one of the key reasons for the limitation of invading T cell activity. Therefore it inhibited T cell mitochondrial activity and resulted in progressive T cell failure, i.e. non-PD-1-dependent mechanism induced T cell depletion. In this case, only eliminating the immunosuppression caused by PD-1/PD-L1 and CTLA-4 can scarcely reach the therapeutic needs, while 4-1BB is a co-stimulating molecule highly expressed on failing T cells [34]. Co-stimulation of 4-1BB induces mitochondrial function and biogenesis in T cells, mainly providing energy for CD8^+^ T cells to increase by activating glucose and fatty acid metabolism [35], enabling cancer immunotherapy response. This non-PD-1-dependent escape can be treated with 4-1BB agonist, and 4-1BB monoclonal antibodies have been applied and tested in clinical trials. However, most clinical trials have indicated that antibodies against 4-1BB agonists have little impact on their own, but are considerably more successful when combined with other immunotherapies [36, 37]. This implies that individual and combination immunotherapy will progressively take over as the primary techniques of immunotherapy.

### 3.2. Cancer Vaccine Combined with Immunotherapy

**Figure 3:**
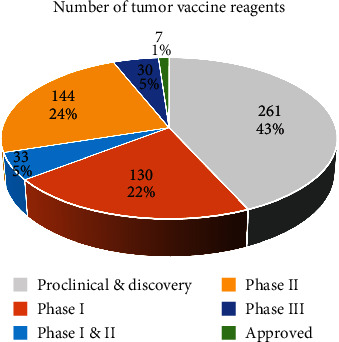
Tang and associates summarized the number of tumor vaccines in each clinical trial phase as of 2018 failed tumor vaccine formulations accounted for 43.1 percent of all clinical trials; only 1.2 percent of tumor vaccine formulations were approved for marketing; most cancer vaccine formulations are in the early stages of clinical trials (Phase I/II).

As one of the effective methods of immunotherapy, tumor vaccination has long been a “hot topic” in tumor treatment strategy research. The statement regarding vaccine availability in the commercial and experiment field and its failure rate has been summarized by Tang et al. [38] in 2018 (Figure 3). Since the therapeutic tumor vaccines have a high failure rate, most of them are still in the preclinical stages. Antigens, which cause acquired immunity and immune cells and antibodies (T cells, DCs, IgG, and so on) that induce acquired immunity, are the focus of tumor vaccine providers [2, 39, 40]. However, as the disease progresses, limited studies have been conducted to determine if mainly activated immune cells such as T cells and APCs can combat immunological escape in the TME. This might play a significant role in the failure of therapeutic cancer vaccinations.

#### 3.2.1. Tumor Vaccine Combined with PD-1/PD-L1 Antibody

Tumor vaccines can boost T cell activation and immunological response however, tumor vaccines alone can induce tumor escape. Shi and colleagues [41] discovered that following tumor vaccine administration, PD-1 is substantially expressed, and the PD-1/PD-L1 pathway remains open, suppressing T cell immunity and encouraging tumor escape. As a result, combining a tumor vaccination with PD-1/PD-L1 antibody treatment can have a more potent antitumor immunological impact, increasing immune cell lethality and weakening immune suppression. T-lymphocyte infiltration is lower in immunological “cold” tumors, and immunotherapy is less effective [42], which may explain why PD-1/PD-L1 blockers do not demonstrate substantial advantages in PCa. Mc-Neel and colleagues [43] demonstrated the effectiveness of DNA vaccination in combination with PD-1 blockers in the treatment of PCa. Although there was no proven complete response/partial response (CR/PR), tumor volume was considerably decreased in 4/5 patients after 12 weeks of concurrent therapy, and PSA was lowered from baseline.

**Figure 4:**
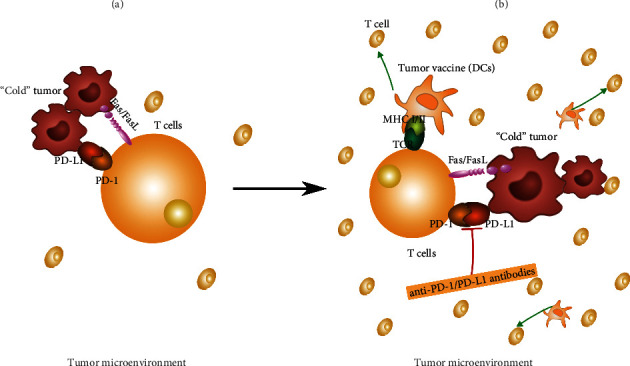
Mechanism of combination immunotherapy in the treatment of immune “cold” tumors. (a) Microenvironment of an immune “cold” tumor before immunotherapy: less inflammatory cell infiltration and less lethal. (b) Changes of the TME after the combination of DCs tumor vaccine and ICIs inhibitor in the treatment of immune “cold” tumors: DCs tumor vaccine can enhance T cell infiltration, and PD-1/PD-L1 inhibitors can block the activation of PD-1/PD-L1 pathway and prevent T cell inactivation.

The major aim for immunological “cold” tumors is to increase T cell infiltration while simultaneously considering immune escape, i.e., in conjunction with PD-1 antibodies, as illustrated in Figure 4 This appears to give a better direction for cold tumor immunotherapy and becomes one of the possible tumor vaccine indications when coupled with ICIs. This implies that combining a tumor vaccination with ICIs is a potential therapeutic strategy.

#### 3.2.2. DCs Vaccine Combined with Immune Adjuvant

DCs mainly centralized on immune cell research to create cancer vaccines, with Sipuleucel-T being the first DCs-based vaccination licensed for PCa [44]. It primarily failed to establish clinical effectiveness [45], i.e., a slightly increased median survival in the lack of evidence of long-term progression-free survival [9], and is now being investigated as combination immunotherapy. Preconditioning DCs with immune adjuvants Flt3L and TLR3 has been shown in studies to successfully increase DC aggregation, tumor antigen-specific CD8^+^ T cell proliferation, and antitumor immunological impact [46, 47]. Hensel and colleagues [48] showed that a tumor vaccination coupled with an adjuvant can break immune suppression and induce an antitumor response. Clinical studies of DC-based vaccinations coupled with Flt3L and TLR3 agonists are underway (NCT01976585) [49].

The simultaneous application of the tumor vaccine is primarily to minimize the immunosuppressive impact of the TME to enhance the tumor vaccine's antitumor immunological action. At present, there is no approved treatment method of tumor vaccine combined with other immunotherapies, but the clinical trials of tumor vaccine combined with other immunotherapies are being carried out in succession (see Table S2).

### 3.3. Individualized Therapy Targeting Neoantigens

The combination of the PD-1/CTLA-4 antibody has strongly affected tumor treatment. However, it still has a significant resistance to this therapy in critical organ malignancies such as liver cancer and pancreatic cancer, showing severe tumor specificity. TNAs are highly personalized and originate from random non-synonymous mutations produced by DNA mismatch repair in tumor cells [50]. The frequency of non-synonymous mutations is significantly greater during tumor development [51]. Vogelstein and colleagues' [52] research suggest that cancer mismatch repair errors can selectively up-regulate immunosuppression checkpoints such PD-1/PD-L1, CTLA-4, and IDO, resulting in local immunosuppression and TNA aggregation. Because TNAs do not present in healthy tissues, thymus selection and central/peripheral tolerance do not apply [53]. TNAs have been proven in studies to be useful as immunological targets in treating solid malignancies [54]. As a result, TNAs may be an ideal immunological target. There is a wonderful opportunity to create immunotherapy for TNAs with exact molecular features to achieve the possibility of customized immunotherapy.

#### 3.3.1. Individualized T Cell Therapy

Stevanovic and colleagues [55] used targeted neoantigen-T cells to treat metastatic cervical cancer. Tumor regression and no trend of in vivo proliferation in cancer patients recommended that researchers investigate and create neoantigen-T cell treatment. Although neoantigen mutations are personalized, ACT targets TNAs [56]. The use of neoantigen-specific T cell immunotherapy necessitates the acquisition of neoantigen-specific T cell populations or TCRs. Ali and colleagues [57] demonstrated that DCs were transfected with mRNA encoding potential novel epitopes. The DCs then stimulated T cell populations in healthy individuals to generate neoantigen-specific T cells. According to Li and colleagues [58], target cell morphogenesis is an effective method for screening neoantigen TCR ligands. The crucial issue is obtaining a large number of particular T cells. Many researchers are investigating various techniques; however the study is still in its early stages, and there are few effective solutions for clinical use.

#### 3.3.2. Individualized Tumor Vaccine Therapy

With the fast advancement of genomics and bioinformatics, it is now feasible to individually examine the neoantigen gene sequence produced by tumor mutations [59]. To optimize the immunological response of T cells targeting the novel epitopes, the best-predicted TNAs from somatic mutant lines must be screened. Because Spontaneous mutations cause tNAs, MHC-I/II restricted neoantigens can be utilized to create tumor-specific vaccinations. Previously, TNA screening was mostly focused on CD8^+^ T cell identification of MHC-I restriction neoantigens. Nonetheless, the involvement of CD4^+^ T cells and MHC-II restriction neoantigens in tumor immunotherapy has lately received more attention. Kreiter and colleagues [60] discovered that MHC-II preferentially binds mutant peptides rapidly and that CD4^+^ T cells, the major immune response, identify particularly altered peptides in both colon cancer and breast cancer animal models. Furthermore, numerous clinical trials have demonstrated that patients retain active CD4^+^ T cells in response to neoantigens even when MHC-I class binding is utilized to predict the neoantigen sequence [61, 62]. Ott et al. [62] clinical study of a customized long-peptide vaccine for melanoma including MHC-I/II limited neoantigen epitopes revealed that four of the six vaccinated patients had no recurrence 25 months after immunization and had significant T cell-specific responses. Tondini and colleagues' DNA vaccine [63] may contain a range of novel epitopes and have been demonstrated to elicit a robust neoantigen-specific immune response in various CD8^+^ and CD4^+^ T cells. These findings imply that the neoantigen vaccination is a viable therapeutic option.

Personalized treatment for neoantigen vaccines is still in its early stages, and numerous issues must be addressed. Predicting the ideal neoantigen gene sequence, for example, is restricted, which may prevent the tumor vaccination from exerting the maximal antitumor impact and contribute to the tumor's treatment resistance developing more quickly.

#### 3.3.3. Individualized Pharmacogenomic Biomarkers Therapy

With the development of genome sequencing technology, researchers found that the process of tumor growth is accompanied by gene mutations, mainly from host cells (germline mutations) and tumor cells (somatic mutations) [64], along with these two mutations are the main reason for the individual differences in the efficacy of antitumor drugs. Pharmacogenomics is the study underlying individual differences in human medication action from the perspective of genes, intending to provide optimal drug doses, avoid unpleasant effects, and allow patients to get the optimum therapeutic benefit possible [65]. Therefore, understanding the molecular characteristics of patients tumors and determining their relationship with drug efficacy is very important to identify predictive biomarkers and provide a basis for individualized treatment.

Somatic mutation provides a basis for individualized, targeted therapy of tumors. Ou et al. showed that patients with non-small cell lung cancer with ALK rearrangement have a significant curative effect on the use of crizotinib [66, 67]. Predictive detection of biomarkers such as ALK can reduce unnecessary treatment for unresponsive patients and help to avoid the potentially toxic effects of treatment. At present, molecularly targeted drugs such as gefitinib are gradually replacing chemotherapeutic drugs and becoming the first-line treatment of tumors [68]. Germline drug genomic markers can identify patients at the highest risk of serious adverse events. Ingle et al. [69] found through gene sequencing that four single nucleotide polymorphisms (SNPs) were similar to the T cell leukemia 1A (TCL1A) gene, which was related to musculoskeletal adverse events in patients receiving adjuvant aromatase inhibitors (AIs). In addition, the mechanism of drug resistance may be found through germline mutation research, which will open up new ideas for clinical treatment [70].

The main limitation that tumor pharmacogenomics is not widely used in clinics is that it is difficult to accurately determine the utility of identified markers/strategies for patients and medical systems. The possibility of prospective clinical trials based on pharmacogenetics guidance is limited.

#### 3.3.4. Individualized Tumor Vaccine Combined with Immune Checkpoint

Immune checkpoints are strongly connected to tumor nonsynonymous mutant peptides, and studies have revealed the presence of neoantigens in patients' blood following treatment with ICIs [71]. Clinical experiments conducted by Le and colleagues [72] investigated the link between PD-1 and tumor mismatch repair faults. The results revealed that the PD-1 antibody had a more significant tumor impact in cancer patients with mismatch repair errors. This indicates that the combination of immunological checkpoints and particular neoantigen-targeted therapy in the follow-up treatment may be explored, and the impact can be considerably boosted, as demonstrated in clinical studies. PD-1/PD-L1 inhibition coupled with tumor-specific T lymphocytes can prolong animal life in a mouse model of pancreatic ductal carcinoma (PDA) [73]. Ott and colleagues' [62] clinical trial of an individualized vaccine against neoantigens for melanoma revealed that in a clinical trial of six melanoma patients vaccinated with neoantigens, disease progression occurred in two patients, followed by complete tumor regression after anti-PD-1 treatment. The results indicated that the combination of the neoantigen vaccination and ICIs alleviated the tumor's immunosuppression, activated specific T cells, and improved the antitumor immunological response. Tondini et al. [63] created a personalized DNA vaccination with several epitopes that demonstrated synergistic antitumor effects when used with PD-1 inhibitors. The immunotherapy impact is attenuated in immunological “cool” tumors due to low neoantigen mutant peptide and T cell infiltration. However, neoantigen immunotherapy appears to be a breakthrough. The use of a neoantigen tumor vaccination in treating glioblastoma patients resulted in specific T cell activation [61]. A neoantigen tumor vaccination induced specific T cell activity in patients with glioblastoma, but the T cell immunological response was reversed.

**Figure 5:**
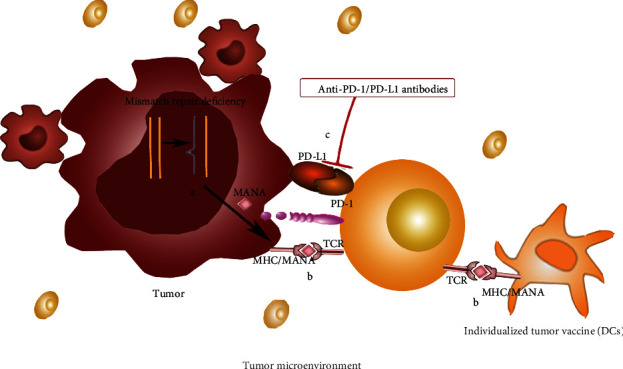
Mechanism of individualized tumor vaccine combined with ICIs in the treatment of tumors. (a) Tumor cells undergo DNA-specific mutations that produce neoantigens. (b) Specific individualized tumor vaccines enable T cells to express specific TCR receptors that recognize neoantigens, thus enabling T cells to target tumor tissues. (c) Combined with the PD-1/PD-L1 antibody, the activation of the PD-1/PD-L1 pathway was blocked. This prevents the inactivation of tumor-specific T cells that target neoantigens.

It was proposed that a tumor vaccination might be utilized to stimulate particular T cell activity. It was required to keep T cell activity from being suppressed and depleted. To improve antitumor efficacy, ICIs might be combined. Anti-CTLA-4 therapy dramatically increases tumor-specific T cell responses in patients with metastatic castration-resistant PCa (mCRPC), which is also an immunological “cold” tumor [74]. This opens the door to a novel immunotherapy approach for immunological “cold” cancers (Figure 5).

In clinical studies, neoantigen-targeted tumor immunotherapy has produced outstanding outcomes and has become a “hot” treatment. However, there are concerns regarding whether cancer causes immune escape. Verdegaal and colleagues [12] demonstrated that neoantigen expression was dynamically altered, and that neoantigens identified by T cells may be selectively eliminated from tumor cell populations. Balachandran and colleagues [75] discovered that infiltrated T cells in patients with pancreatic ductal adenocarcinoma who seemed to have a relatively long survival time had a sustained high response to the selective loss of neoantigens that occurred in patients with metastasis, implying neoantigen immunoediting. As a result, tumor vigilance may evade T cell immune surveillance by decreasing the expression of known neoantigens.

### 3.4. Immunotherapy for MHC Deficiency

MHC deficiency is indeed a dilemma in T cell immunotherapy, as ICIs and tumor vaccines can only be effective if tumor antigens are recognized by T cells. Regular production of MHC molecules is required for T cells to detect specific antigens and play an immunological function, and malignancies would definitely evade immune surveillance if MHC expression is reduced. Low MHC expression, on the other hand, is now a key pathway for tumor escape, and there is presently no effective therapy. MHC deficiencies are typically classified as reversible or irreversible [76], and their therapy differs accordingly. This review focuses on the most recent immunotherapy studies for cancers with minimal MHC expression as well as novel immunotherapy techniques which may be successful.

#### 3.4.1. Treatment of MHC Reversible Defects

MHC deficiency is indeed a dilemma in T cell immunotherapy, as ICIs and tumor vaccines can only be effective if T cells recognize tumor antigens. T cells must regularly produce MHC molecules to detect specific antigens and play an immunological function. Malignancies will evade immune surveillance if MHC expression is reduced. On the other hand, low MHC expression is now a key pathway for tumor escape, and there is presently no effective therapy. MHC deficiencies are typically classified as reversible or irreversible [76], and their therapy differs accordingly. This review focuses on the most recent immunotherapy studies for cancers with minimal MHC expression and novel immunotherapy techniques that may be successful.

#### 3.4.2. Treatment of Irreversibly Defective MHC Tumors

**Figure 6:**
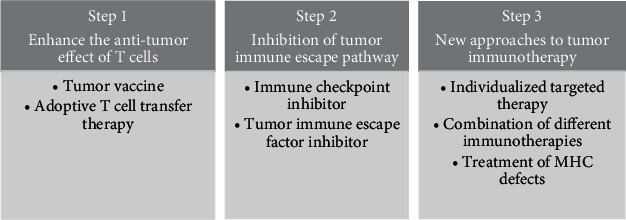
The gradual improvement of tumor immunotherapy strategies. In Step 1, the main goal is to enhance the lethality of the immune system against the tumor to reduce the harm of tumors spread to the human body. In Step 2, the researchers found that cancer could escape from the monitoring of the immune system through a series of immune pathways, from merely increasing the T cell effect to suppressing the tumor immune escape pathway. In Step 3, to further enhance the response rate and overall survival of the tumor patients, a new method of immunotherapy based on combination therapy and individualized therapy was adopted. MHC expression deficiency plays an essential role in tumor immunotherapy, one of the main directions for future treatment.

The therapy of cancers with reversible MHC deficiencies has obvious limitations, particularly when it comes to permanent alterations like genetic loss and mutations. To do so, we must develop novel therapies. Barkal and colleagues [77] discovered another important role for the signal axis MHC-I/LILRB1. When MHC expression was reduced, the inhibitory receptor LILRB1 on the surface of macrophages lost its function, and tumor cells became more sensitive to macrophages. Cancer cells activate the CD47/SIRP axis as an antiphagocytic signal to prevent macrophage-mediated demise. Treatment that inhibits CD47 and SIRP can significantly boost the antitumor response generated by macrophages [78]. CD47 and SIRP antibodies are now being tested in clinical studies as immunotherapy methods. According to the newly found signaling axis, low MHC-I expression may be utilized as a possible biomarker, and the response effect of patients treated with anti-CD47 and anti-SIRP may be greatly enhanced. Van Hall and colleagues [79] discovered novel cytotoxic T lymphocytes (CTLs) that can be utilized to treat irreversibly low-expressed MHC tumors caused by a lack of the transporter associated with antigen processing (TAP). TAP's low expression will develop a distinct antigen epitope, similar to Lass5 protein, leading CTLs to self-screen for specialized CTLs targeting the new antigen. If an epitopes library of TNAs with MHC defects was developed and based on the sequence of TNAs with irreversible MHC deficiencies found in patients, the therapeutic impact of customized therapy neoantigen-targeted tumor vaccines or ACT might be dramatically improved. Karre et al. [80] suggested the deletion of self-hypothesis that MHC-I molecule deletion would cause NK cell cytotoxicity. Recent research, however, has placed this hypothesis into consideration, claiming that down-regulation of MHC-I molecules does not result in NK cell autoreactivity [81]. This might be due to an interesting phenomenon found by the researchers, in which mature NK cells can establish their phenotype and reactivity in response to changing MHC-I environments [82], and even privileged NK cells in MHC deficiency would result in immunological tolerance. The particular mechanism is unknown, although competent NK cells have more active receptor NKp46 and suppressed receptor Ly49A compared to naïve NK cells [83]. CAR-NK cell treatment has been proven in ovarian cancer xenotransplantation models to dramatically increase antitumor effects [84]. When T cell treatment fails in MHC-I defective malignancies, NK cell immunotherapy coupled with anti-CD47 and SIRP antibodies can be utilized to increase tumor cell sensitivity to macrophages and improve the destructive effect of NK cells. Bern and colleagues [81] demonstrated that NK cells' self-deficiency tolerance is disrupted in inflammatory conditions. As a result, maintaining an inflammatory milieu is critical for promoting DC maturation and, breaking the tolerance environment, improving immunotherapy. Regardless of the therapeutic approaches mentioned above, we should know the tumor immune escape route, such as M2 macrophage polarization, NK cell maturation suppression, and neoantigen selective expression in cancers. To select the next treatments, the likelihood of tumor immune escape should be estimated. The combination application might be chosen to diminish tumor tolerance; nevertheless, the specific efficacy must be confirmed. According to the prior explanation, immunotherapy for tumors is constantly improving (Figure 6).

## 4. Clinical Treatment-Related Adverse Events

This study focuses on a new approach to tumor immunotherapy - combined application and individual therapy - proven clinically meaningful through clinical trials or mechanism verification. However, as we all know, there are inadequate responses to every treatment, which is one of the major issues limiting the new method's widespread application.

### 4.1. Adverse Reactions Related to Combined Immunotherapy

To promote immunological tolerance in vivo, checkpoints such as PD-1, PD-L1, CTLA-4, and IDO are essential. Therefore, ICIs may impair the immune tolerance process, leading to immune-related adverse reactions as Grover's disease and autoimmune colitis [17, 18]. ICIs are typically utilized in conjunction with T cell immune effect enhancement in novel tumor immunotherapy methods to prevent T cell inactivation. Combination immunotherapy offers certain advantages from a mechanistic standpoint, but it exacerbates severe immune-related side effects. The FDA has approved the use of PD-1/CTLA-4 antibody to treat melanoma however the toxicity of its immune-related adverse effects cannot be overlooked. The use of the PD-1/CTLA-4 antibody in combination can successfully cure PCa, however, it has not been authorized due to severe side reactions (2019). In response to this issue, Perez-Ruiz and colleagues [85] suggested that using TNF inhibitors as a preventative measure might minimize the occurrence and severity of adverse responses to dual blocking immunotherapy with CTLA-4 and PD-1. TNF medicines, such as infliximab and adalimumab, are already recognized to treat autoimmune disorders, though further research is needed to determine whether immune-related adverse effects might be a possible indication for combination usage. Different dosages or regimens of nivolumab and ipilimumab were also discovered to have greater effectiveness and less toxicity for diverse tumor types [86]. This might be a breakthrough that permits people to tolerate immunotherapy and warrants further research. The combination use of the PD-1 antibody and tumor vaccination is still in the early stages of clinical trials. Mc-Neel and colleagues [43] performed a clinical study of pembrolizumab combined with an individualized tumor vaccination in 26 patients with progressing mCRPC. There were grade 2 and 3 adverse events and diarrhea, thyroid dysfunction, and hepatitis; no grade 4 adverse events were discovered. And all of these instances were thought to be connected to pembrolizumab. This can be accomplished by adhering to the criteria for hazardous therapy associated with immune checkpoint inhibitor side effects. We should pay great attention in the follow-up clinical trial to see whether there will be any serious adverse effects.

### 4.2. Adverse Reactions Related to Individualized Immunotherapy

Individualized immunotherapy is primarily in preclinical studies at the moment. From a perspective of mechanism, accessible personalized immunotherapy targeting tumor tissues can reduce harm to unaffected tissues. It will generate relatively minor side effects. Ott et al. [62] report that mild influenza-like symptoms, injection site responses, rash, and tiredness were among the treatment-related adverse events in six melanoma patients treated with a customized tumor vaccine. Symptomatic therapy is an alternative way to treat. There were just a few patients in the clinical study. Additional clinical trials are needed to properly monitor additional related severe adverse events.

## 5. Conclusion and Perspectives

The tumor immune microenvironment system is complicated and intertwined, and it is heavily reliant on the contradiction between the normal immune system and the malignant condition of the tumor. Tumor features cannot be generalized; they will differ depending on the individual and tumor type or subtype; hence, individualized tumor immunotherapy is critical. Tumor cells, on the other hand, are tricky, and they will selectively express important targets based on the treatment method used to complete immune escape. As a result, combining immunotherapy can minimize the risk of tumor tolerance. It is essential to adapt therapeutic approaches to understanding the medication action pattern and drug resistance mechanism to create a combination therapy that minimizes antagonism.

Nonetheless, the tumor's complexity should not be underestimated. Immunotherapy should be a never-ending battle against tumor evasion. As a result, successive immunotherapy in combination treatment can be explored. Various combinations of immunotherapy are employed sequentially based on the individual tumor mutation burden, immune checkpoint expression, MHC expression, and other indications. A few trials have been conducted on sequential treatment utilizing immunotherapy individually [87]. Combination immunotherapy is very individualized and should be researched and verified further.

## Figures and Tables

**Table 1:** Completed clinical trials of combining immunotherapy.

ClinicalTrials.gov identifier	Phase	Treatment arms	PFS (month)	ORR (month)
NCT01454102	I	4 doses of Niv/Gem/Cis (*n* = 12) vs. Niv (*n* = 52) vs. Niv/Erlotinib (*n* = 21) vs. 4 doses of Ipi/Niv followed by Niv (*n* = 25)	50.5 (18.7 to 75.7) vs.50.6 (27.7 to 69.7) vs.39.7 (26.0 to 53.1) vs.72.4 (54.7 to 84.1)	41.7 (15.2 to 72.3) vs.23.1 (12.5 to 36.8) vs.19.0 (5.4 to 41.9) vs.47.4 (31.0 to 64.2)
NCT01927419	II	4 doses of Ipi/Niv followed by Niv (*n* = 95) vs. 4 doses of Ipi/Pla followed by Pla (*n* = 47)	8.57 (7.03 to NA^[1]^) vs. 3.73 (2.76 to 5.13)	59.7 (47.5 to 71.1) vs. 10.8 (3.0 to 25.4)
NCT02905266	III	4 doses of Ipi/Niv followed by Niv (*n* = 53) vs. 4 doses of sequential administration of Niv and Ipi Q3W followed by Niv (*n* = 53)	10.25 (2.96 to NA^[1]^) vs. NA (4.96 to NA)	52.8 (38.6 to 66.7) vs. 60.4 (46.0 to 73.5)

## Data Availability

The authors confirm that the data supporting the findings of this study are available within the article and its supplementary materials.

## Supplementary Materials

Supplementary Materials Table S1: clinical trials of combined application of CTLA4 antibody and PD-1 antibody. Table S2: clinical trials of tumor vaccines combined with other immunotherapies.
