# Supplementary material for: Understanding of Immune Escape Mechanisms and Advances in Cancer Immunotherapy
Source: J Oncol. 2022 Mar 31;2022:8901326. doi: 10.1155/2022/8901326 (PMC8989557; doi:10.1155/2022/8901326)
Supplement: Supplementary Materials — Table S1: clinical trials of combined application of CTLA4 antibody and PD-1 antibody. Table S2: clinical trials of tumor vaccines combined with other immunotherapies. [file 8901326.f1.pdf]

Table S1. Clinical trials of combined application of CTLA4 antibody and PD-1antibody.

NCT identifier	Brief title	Disease condition	Therapeutic interventions	Phase	Status as of March 2020
NCT02917772	Tailored immunotherapy approach with nivolumab in subjects with metastatic or advanced renal cell carcinoma.	Metastatic or advanced renal cell carcinoma	Nivolumab plus Ipilimumab	II	Active, not recruiting
NCT02939300	Ipilimumab and nivolumab in leptomeningeal metastases.	Leptomeningeal metastases	Nivolumab plus Ipilimumab	II	Active, not recruiting
NCT03241186	Ipilimumab and nivolumab as adjuvant treatment of mucosal melanoma.	Mucosal melanoma	Nivolumab plus Ipilimumab	II	Recruiting
NCT03297593	Nivolumab, in combination with ipilimumab in patients with metastatic renal cell carcinoma.	Renal cell carcinoma	Nivolumab plus Ipilimumab	II	Recruiting
NCT03305445	Nivolumab/ipilimumab-primed immunotransplant for DLBCL.	Relapsed refractory diffuse large B-cell lymphoma	Nivolumab plus Ipilimumab	II	Recruiting
NCT03246958	Nivolumab plus ipilimumab in thyroid cancer.	thyroid cancer	Nivolumab plus Ipilimumab	II	Recruiting
NCT03219671	Nivolumab and ipilimumab in classical Kaposi sarcoma (CKS).	Classic Kaposi's sarcoma	Nivolumab plus Ipilimumab	II	Recruiting
NCT03172624	Study of nivolumab plus ipilimumab in patients with salivary gland cancer.	Salivary gland cancer	Nivolumab plus Ipilimumab	II	Recruiting
NCT03510871	Nivolumab plus ipilimumab as neoadjuvant therapy for hepatocellular carcinoma (HCC).	Hepatocellular carcinoma (HCC)	Nivolumab plus Ipilimumab	II	Not yet recruiting
NCT04090710	SBRT with combination ipilimumab/nivolumab for metastatic kidney cancer.	Metastatic renal cell carcinoma	Drug: Ipilimumab Nivolumab Radiation: SBRT+ Ipilimumab/Nivolumab	II	Recruiting
NCT0391	Durvalumab and	Somatically	Durvalumab plus	II	Recruiting

1557	tremelimumab combination in somatically hypermutated recurrent solid tumors.	hypermutated recurrent solid tumors	Tremelimumab

Source: clinicaltrials.gov.

Table S2: Clinical trials of tumor vaccines combined with other immunotherapies.

**NCT identifier**	**Brief title**	**Condition**	**Intervention**	**Phase**	**Status as of March 2020**
**NCT03121677**	Personalized tumor vaccine (PCVs) strategy and PD-1 blockade in patients with follicular lymphoma.	Follicular lymphoma	Biological: Personalized tumor vaccine<br>Biological: Nivolumab<br>Biological: Rituximab	I	Recruiting
**NCT03525652**	Therapeutic vaccine plus PD-1 knockout in PCa treatment.	PCa	Biological: Therapeutic vaccine<br>Biological: PD-1 Knockout T Cells	I/II	Recruiting
**NCT04161755**	Study of PCVs and a PD-L1 blocker in patients with pancreatic cancer That can be treated with surgery.	Pancreatic cancer	Drug: Atezolizumab<br>Biological: RO7198457<br>Drug: mFOLFIRINOX	I	Recruiting
**NCT03897881**	An efficacy study of adjuvant treatment with the PCV mRNA-4157 and pembrolizumab in patients with high-risk melanoma (KEYNOTE-942).	Melanoma	Biological: mRNA-4157<br>Biological: Pembrolizumab	II	Recruiting
**NCT04201873**	Pembrolizumab and a vaccine (ATL-DC) for the treatment of surgically accessible recurrent glioblastoma.	Recurrent glioblastoma	Biological: Dendritic Cell Tumor Cell Lysate Vaccine<br>Biological: Pembrolizumab	I	Recruiting
**NCT04096911**	Combination of PD-1 monoclonal antibody and HPV vaccine in patients with cervical cancer.	Cervical cancer	Drug: Sintilimab<br>Drug: quadrivalent HPV vaccine	II	Recruiting
**NCT04117087**	Pooled mutant KRAS-targeted long peptide vaccine combined with nivolumab	Colorectal cancer<br>Pancreatic	Drug: KRAS peptide vaccine<br>Drug: Nivolumab plus Ipilimumab	I	Not yet recruiting

	and ipilimumab for patients with resected MMR-p colorectal and pancreatic cancer.	cancer			
**NCT04246671**	Phase 1/2 trial of intravenous TAEK-VAC-HerBy vaccine alone and in combination treatment in HER2 cancer patients.	Breast cancer	Biological: TAEK-VAC-HerBy<br>Biological: HER2 antibodies	I/II	Not yet recruiting

Source: clinicaltrials.gov.
